# Flexural Tests for Efficiency Evaluation of Spike Anchors on CFRP-Strengthened Concrete

**DOI:** 10.3390/ma16010164

**Published:** 2022-12-24

**Authors:** Paula Villanueva Llauradó, Rafael Cascón Porres, Alberto Sanchidrián Blázquez, Francisco Santos Olalla, Fernando Gómez Álvarez

**Affiliations:** 1 Departamento de Estructuras y Física de Edificación, ETSAM, Universidad Politécnica de Madrid (Technical University of Madrid, UPM), 28040 Madrid, Spain; 2 Departamento de Ingeniería Mecánica, Química y Diseño Industrial, ETSIDI, Universidad Politécnica de Madrid (Technical University of Madrid, UPM), 28012 Madrid, Spain

**Keywords:** FRP-to-concrete bond, anchorage system, spike anchors, bond length

## Abstract

Spike anchors are one of the most promising techniques to prevent or delay debonding in FRP reinforcement sheets. There are several parameters affecting the anchors’ capacity, such as the embedment length and dowel angle. Regardless of the anchors’ capacity, their contribution to the overall strength of the anchored joint is affected by a larger number of variables, including the bonded length behind the anchors, the number and arrangement of the anchors, and the contact surface between the anchor fan and the FRP sheet. This paper presents experimental results of 10 tests conducted on concrete beams. In the tests, anchored joints reached peak loads up to 155% of those of unanchored, bonded joints. The main finding of the research is that the bond length in front of and behind the anchors affects both the peak load and the overall behaviour, with unbonded anchored joints exhibiting a poor behaviour and premature slippage of the anchor, without achieving its failure due to fibre rupture.

## 1. Introduction

Fibre-reinforced polymers (FRPs) are broadly employed as a retrofitting method for existing reinforced concrete structures, due to the considerable advantages of these materials, such as their high strength-to-weight ratio [1,2]. However, one limitation of this technology is its premature debonding failure from the substrate. This failure mode has been widely studied [3,4,5] and is the basis for design models in different international guidelines [6,7,8]. Additionally, international researchers have aimed to develop anchorage techniques which are able to prevent of delay debonding [9,10,11]. Such techniques mainly include C-Anchors [12], patch anchors [13], and spike anchors. Among these techniques, FRP spike anchors (in this paper, “spike anchors”, “FRP anchors”, “anchors” and “connectors” will be used indistinctively) have been highlighted as they are versatile, non-corrosive, and have more ease to control during application [14,15]. Previous studies on FRP anchors employed handmade solutions consisting of a rolled sheet, in which one half was embedded in the substrate by inserting it into a predrilled hole, and the other half conformed and a splay region, of anchor fan, which was bonded onto the FRP reinforcement. In subsequent research works, anchors were developed from carbon fibre ropes, keeping the two distinct regions. Aiming at further industrialization, the dowel part can be precured with an epoxy resin matrix, so it can be easily introduced in the predrilled hole in the substrate. 

In recent years, concern has arisen related to the poor FRP performance in the event of fire or elevated temperatures, as collected in different papers, such as [16,17,18]. This is another motivation for the employment of anchors, for instance in preinstalled sheets that may have suffered adherent loss due to high temperatures. However, it should be noted that FRP anchors do employ epoxy resin in their contact with the FRP sheet, which must be protected similarly to the reinforcement against high temperatures.

The potential performance improvement of FRP sheets with spike anchors has been broadly highlighted in literature. However, lack of knowledge regarding some parameters affecting the performance of anchored joints has hindered their inclusion in international codes and design guidelines. The main results and analytical models for spike anchor’s behaviour have been collected in a recent review by Muciaccia et al. [19], indicating important differences and limitations in the available models. To date, three main approaches have been followed in research studies on FRP anchors to date: tests on isolated connectors, test on anchored joints and numerical modelling, Within the tests on anchored joints, most studies have employed single shear or double shear tests, while only limited research has been conducted on beam tests.

### 1.1. Tests on Isolated Anchors

Tests on isolated connectors allow identifying certain parameters that affect the strength of the anchors themselves when installed into predrilled holes, such as the embedded length, dowel angle, and bending radius [20,21]. Anchors can be organized according to whether they are subjected to tensile loads (straight anchors) or, more frequently, to shear stresses. When anchors are subjected to shear stresses, a crucial issue is the premature fibre rupture due to kinking in the bending region in the transition between the dowel and the anchor fan. The experimental results on isolated connectors have led to the development of a strength model for anchors that are mainly subjected to tensile stresses [22], object of a recent revision by means of the extension of the database [23], and of a strength model for anchors subjected to shear stresses, especially regarding the failure mode due to stress concentration in the bending region [24].

### 1.2. Tests on Anchored Joints

The second approach comprises a range of experimental studies including single-shear, double-shear tests, and beam tests. In all these cases, anchors are subjected to shear stresses as the surface of the substrate is aligned with the tensile force. Shear tests allowed studying parameters, such as the bonded length in front of the anchor and behind the anchor, also referred to as end length [25,26], the connector-to-reinforcement strength ratio [5] or the influence of multiple anchors [26,27,28]. Based on these results, different predictive models have been developed to date. The goodness-of-fit of each of these models was discussed in Cortez et al. [29] with respect to an extensive database comprising results from different authors. The main limitation of this approach is that the concrete substrate is not subjected to the same stress distribution as in beams, and no bending strains are induced.

### 1.3. Numerical Modelling

Numerical modelling has been used to evaluate the contribution of spike anchors to overall strength of anchored joint [30], but the efforts have been mainly focused on assessment of the numerical models (FEM) through comparison with experimental results [31]. In this sense, it should be noted that numerical modelling can predict different arrangements to those studied, but within the same test type. Given the scarcity of beam tests on anchored FRP sheets, FEM models have been used for correlation with shear tests, with the consequent limitations in terms of strain prior to debonding initiation and propagation, and different stress distribution in the concrete surrounding the anchor dowel. Indeed, as stated in the review paper by Naser et al. [32], future works on numerical modelling of externally bonded FRP-to-concrete beam reinforcement should include modelling the performance of joints with spike anchors. In this sense, the most recent paper covering that topic of FRP anchors in shear tests [31] employs a part for the concrete block, to which boundary conditions are applied to represent the constraints during the shear test (rotation and displacement at two opposite faces, one of them is the tensile force that is applied to the FRP sheet). In this study, the concrete was modelled under the plastic-damage plasticity model in ABAQUS. The other modelled parts were the FRP sheet, a material representing the resin between anchor dowel and concrete block, and the anchor. Modelling of the FRP-to-concrete interface used a cohesive surface interaction, with fracture energy being obtained according to fib-90 [8]. For numerical modelling of bonded, unanchored FRP sheets to concrete beams, further information can be found in Naser et al. [32]. On the other hand, bond of FRP sheets to concrete (without anchors) is being increasingly defined by means of numerical modelling of complex situations. In this sense, fracture mechanics-based approaches (especially those based on CZM) are the most common to widely analyse the nonlinear behaviour of such structures [33,34,35,36].

### 1.4. Beam Tests 

There is a severe scarce of beam tests, which makes it difficult to draw conclusions about whether the adherent joint performance may be affected by the actual orientation of the stresses in beams. Sun et al. [37] conducted two series of three point bending tests with an anchorage system comprising spike anchors and patches, with the aim of minimising the influence of the bonded length behind the anchor (end length); they conducted a special series in which the bonded length was reduced, to test the impact of a degraded bond on the joint strength. They concluded that the use of the patch can allow fuller use of the connector thus minimising the impact of short end lengths, and that the bonded length has a considerable influence on the behaviour of the anchored joint. 

Zaki et al. [38] conducted a series of tests on 4725 mm long beams employing four points beams tests. Tests were performed by comparing the performance of anchored FRP sheets on T-beams and rectangular beams, with a total height of 305 mm. The main observations were that employing multiple anchors in longitudinal arrangement allowed full-flexural capacity of the beams, completely prevented debonding. However, no insights into the anchor’s individual performance of parameters affecting the anchor’s behaviour with respect to the FRP sheet were studied, and the anchors were not designed according to the existing models to maximize connector’s bend strength.

### 1.5. Identification of Main Paramters Affecting Anchored Joint Performance

For narrow sheets with one anchor, the end length has been identified as the most influential parameter affecting the joint performance [25,26]. However, the parameter can be hardly isolated from the overall performance of the anchor and from the influence of geometrical parameters, such as the bending radius or the dowel angle. Additionally, there is a lack of consensus regarding the influence of the adherent strength of the reinforcement-to-concrete interface on the overall strength of anchored joints. Del Rey et al. claim that there are no significant differences between bonded and unbonded anchored reinforcements [14]. On the other hand, the design model by Cortez et al. [29] assumes that the overall strength of bonded, anchored reinforcements is the product of the addition of the bond strength plus the connector’s strength, modified due to the influence of the anchor position. Similarly, Sun et al. concluded from a series of beam tests that a long-term bond loss can compromise the capacity of the anchored FRP sheet [37].

In this study, four series of tests were conducted in order to evaluate the influence of bonded length in front of and behind the anchor on the performance of anchored CFRP reinforcement sheets. Three series comprised a variation of the bonded length behind the anchor fan, while the fourth series was a control series used to compare the results of bonded and unbonded anchored joints. The aim of this research is to complement existing information regarding the influence of bonded length on the overall performance of anchored CFRP joints, while conducting a test that is able to reproduce the actual stressed in reinforced beams by means of including a flexural strengthening. The main significance of the study is the employment of beam tests instead of shear tests, with the aim at evaluating the influence of the deflection of the beam in the anchor’s performance. This motivated the monitorization not only of the sheet, but also of the anchor’s dowel, which is a main novelty with respect to the literature and will allow for better assessment of numerical modelling of complex situations of anchored joints. Additionally, Sun et al. [37] results for partially bonded sheets were complemented with completely degraded interfacial bond behaviour, which allowed observing the noticeably differences in terms of ultimate load and of failure mode between bonded, anchored joints and unbonded, anchored joints. It was concluded that there is a synergistic effect between the anchor and the bond behaviour during bending tests, thus evidencing the problematic of bond loss at low stress conditions.

## 2. Materials and Methods

This section comprises an explanation of the methodology employed in the research, together with the studied materials and parameters. The following flow chart summarises the methodology of the work.

Three-point flexural tests were conducted to determine the interfacial strength of anchored CFRP sheets for concrete retrofitting. As mentioned above, the specimens were designed to study the influence of the bonded length behind the anchor fan on the joint strength, and the general influence of the adherent strength on the anchored joints. The test matrix is collected in Table 1, where the number of anchors is given for a half beam (each beam consisted of two symmetrical halves.

**Figure 1:**
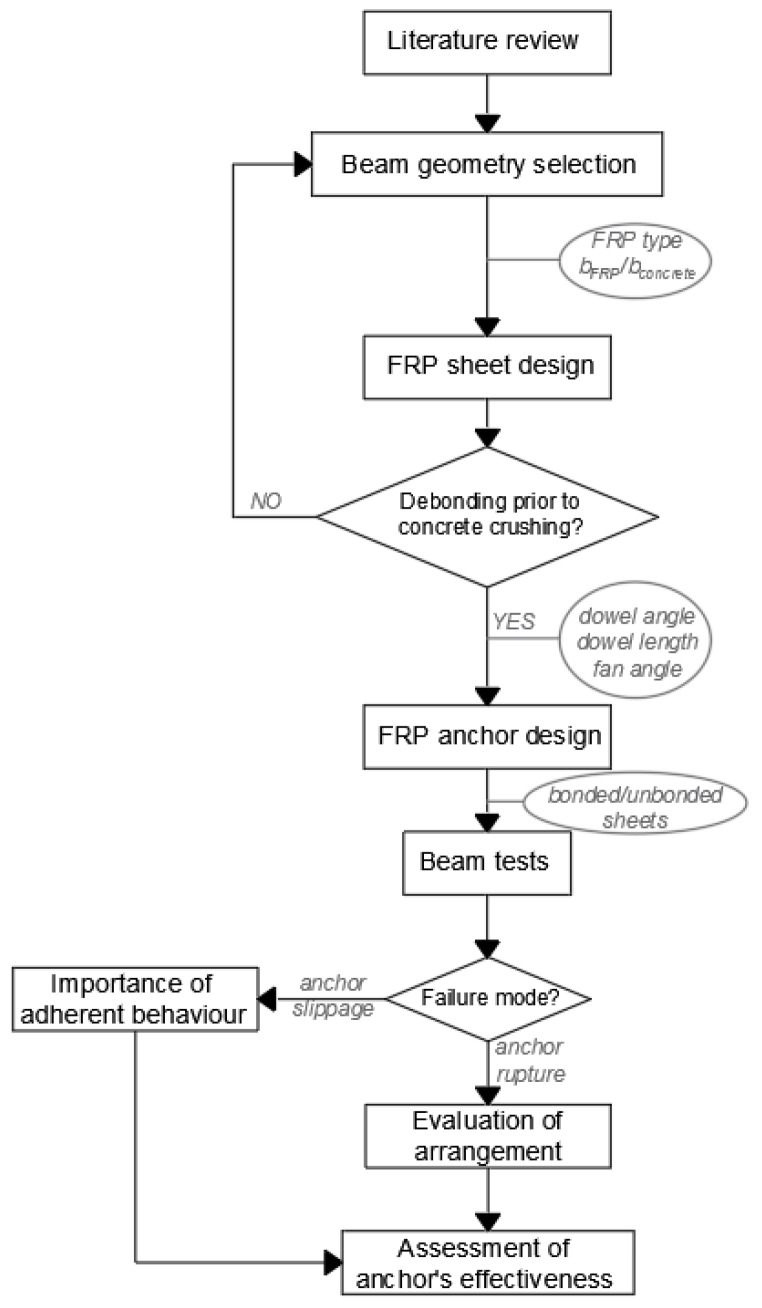
Methodological flow chart.

**Figure 2:**
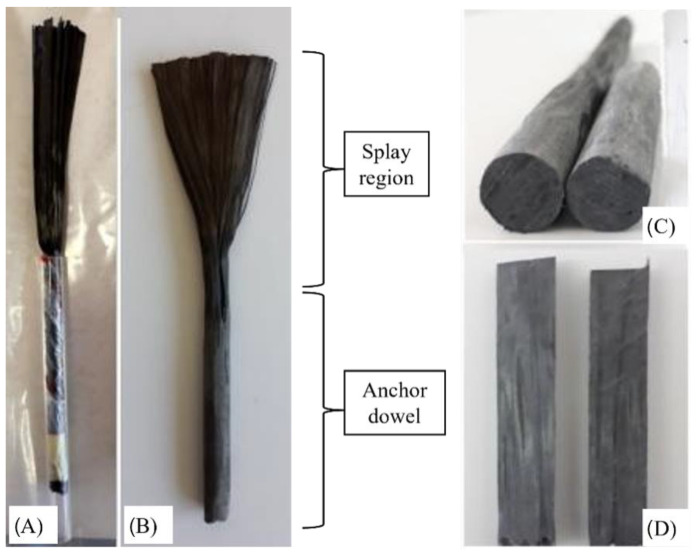
FRP anchor made from carbon rope, with precured anchor dowel. (**A**) Pre-impregnation with epoxy resin; (**B**) Precured anchor; (**C**) Cross section of hardened anchor dowel; (**D**) Inspection of inner impregnation of anchor dowel.

The specimens were 150 × 150 × 700 mm, as shown in Figure 1. Beam geometry was selected for better comparison with previous results by Sun et al. [37]. The three-point tests were performed with a central load and with a span length equal to 600 mm, with a continuous FRP sheet of 500 mm length and 100 mm width. The difference between sheet with and beam width was selected to provide a region outside the edge zones for dissipation of stresses in the substrate. The FRP width was selected among the literature, considering the observations by Cortez et al. [29] that related 50-to-100 mm FRP widths to the best results in terms of spike anchor’s performance. For all specimens, the embedded length of the anchors was 100 mm; anchors with 10 mm diameter were inserted into drillings with a diameter of 20 mm, and with dowel angles of 135°. This configuration in terms of hole-to-dowel clearance and dowel angle was chosen as it was found to provide optimal results in terms of isolated anchors’ capacity in the bending region, according to previous studies [20,24]. Small dowel angles have been related in the literature to premature failure due to fibre kinking provoking fibre rupture in the bending region. On the other hand, angles closer to 180° have been related to concrete cone failure behind the anchor’s insertion point. In [24], dowel angles ranging from 90° to 150° were compared, the best results being obtained for the intermediate values of which 135° is preferable for its ease of control during the drilling. The anchor fan was inserted between two CFRP plies, for all tests; this follows previous findings from comparison of the anchor’s efficiency depending on whether the anchors fan in installed between reinforcement plies or bonded onto the reinforcement [23]. The fan was splayed forming 60° in all cases, covering the whole width of the reinforcement sheets according to the recommendations in [27]; this criterion is widely followed by international researchers on spike anchors. Thence, the overall length of the connectors was 100 mm (anchor dowel) plus 95 mm comprising the bending region and the anchor fan. The anchor type employed in the present research is presented in Figure 2.

**Figure 3:**
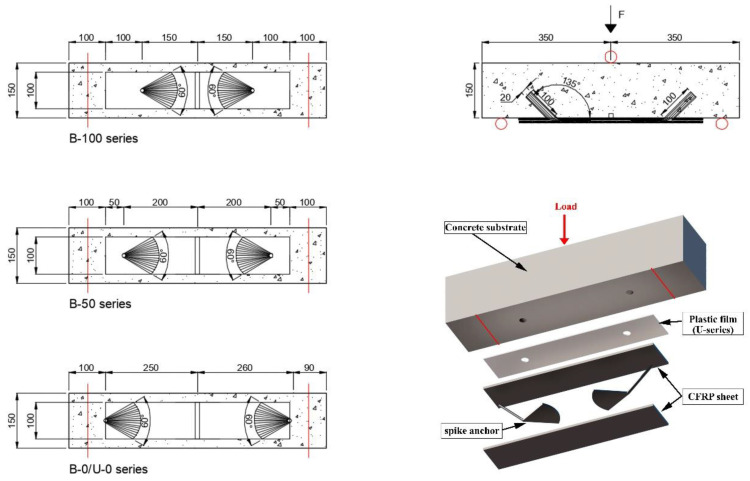
Specimen configuration.

Five different series were evaluated, as collected in Table 1 and Figure 3: a control series (without anchors), three series varying the position of the anchor with respect to the unloaded end of the reinforcement and one series with unbonded, anchored joints, in which anchors were pierced leaving no sheet length behind the anchor (similarly to the B-0 series). The adherence of FRP sheet to the concrete was prevented in the U-series by applying a plastic film between the substrate and the reinforcement, of a material to which the epoxy resin has no adhesion. This led to the load-transfer mechanism being carried entirely by the anchor from the initial load steps.

### 2.1. Material Properties

Concrete from a supply company, with mean compressive strength of 31 MPa (evaluated by means of compressive tests on cylindrical specimens according to UNE-EN-3:2020) was used for all specimens. The classification of the concrete according to Spanish regulation EHE-08 was HA-25/B/IIa, this being equivalent to Eurocode S2 slump class for a XC2 environmental exposure. The following codes were employed for classification: UNE-EN, UNE-EN, resulting in the aforementioned mean compressive strength plus, and slump result of 7 cm. The slump result was obtained with the addition of plasticizer according to UNE-EN 934-2. The employed cement type was CEMI 42.5 R, according to EN 197-1. The aggregates were selected according to UNE-EN, with a maximum aggregate size of 20 mm and aggregate size distribution and characteristics were set according to UNE-EN 933. Water absorption by the aggregates according to UNE-EN 1097-6 was lower than 5% for fine aggregate and 6% for coarse aggregate. Sulphate and chloride content in the aggregates were controlled according to UNE-EN 1744.

**Figure 4:**
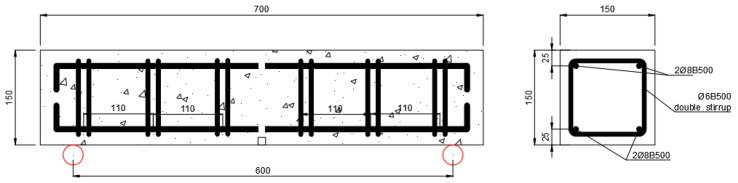
Internal steel reinforcement arrangement.

The Internal reinforcement consisted of B500S steel longitudinal rebars and double vertical stirrups; the longitudinal rebars were not connected in the midspan, nor was the anchorage length provided, to isolate the joint behaviour at each half of the beams. Quality of stirrups was controlled by means of UNE-EN ISO-1. The diameter for bending end hooks of reinforcement was four times the diameter of bent rebar/stirrup. The aim of the internal reinforcement arrangement was to prevent shear failure, while the concrete remains virtually unreinforced against bending moment so as to isolate the performance of the reinforcement. The arrangement of the internal reinforcement can be seen in Figure 4.

A 20 × 20 mm notch was performed at the bottom of the midspan section of the concrete to control the cracking pattern and to assist the isolated behaviour of the two reinforced halves.

Sika Wrap-230C unidirectional carbon fibre fabrics with a nominal width of 300 mm were used as reinforcement. According to the manufacturer, the fibres have an elasticity modulus of 240 GPa, a tensile strength of 4800 MPa and a rupture strain of 2%. The product datasheet specifies a plate thickness with one ply of 0.129 mm, with an elasticity modulus of 220 GPa (average 225 GPa) and a tensile strength of 3200 MPa (average 3500 MPa). On the other hand, tests performed by De Diego et al., revealed that the average elastic module of one ply of the composite is 237 GPa, the average tensile strength is 4161 MPa [39,40]. In the result tables, values in brackets corresponded to the data sheet values. The fabrics were cut to the required width of 100 mm. Anchors were made from carbon fibre ropes SikaWrap FX-50 C, having a diameter of the impregnated connector of 10 mm, According to [41], their fibre cross section is more or equal to 28 mm^2^, and their longitudinal tensile strength is 2100 MPa.

Two different bicomponent epoxy resins were employed: for the impregnation of the anchors and dowel length, Sikadur^®^-52 Injection (Sika SAU, Madrid, Spain) was used, while Sikadur^®^-300 (Sika SAU, Madrid, Spain) was used for the lamination of the reinforcement, FRP-to-concrete bond, and anchor fan-to-reinforcement bond. According to the manufacturer, Sikadur^®^-52 Injection has a tensile strength of 37 N/mm^2^, and an elasticity modulus of 1800 N/mm^2^; Sikadur^®^-300 has a tensile strength of 30 N/mm^2^ and an elasticity modulus of 4500 N/mm^2^.

### 2.2. Specimen Preparation

Concrete was cast in wood open boxes with phenolic treatment, being the bottom face the one to which the FRP reinforcement was applied so that the free surface of the box is the upper face under compression during the test. The formwork fulfilled all requirements in terms of leak prevention, strength for bearing fresh concrete, and for compaction, geometry preservation, and cleanness of the inner panels. The use of first-use phenolic panels allowed demoulding without the need to use chemical products. The concrete curing process was controlled by keeping the upper surface wet in all specimens.

Once the concrete reached 28 days, a precured FRP installation technique was used for all specimens in this research, following the procedure described in [22]. The concrete surface was prepared with diagonal cuts in order to maximise the adherent strength in the FRP-to-concrete interface. In the U-series, no surface preparation of the concrete was performed, and bond between the reinforcement and the substrate was prevented with a plastic film covering the whole length of the reinforcement.

For all the anchored joints, the anchors were installed in predrilled holes which were cleaned according to the available guidelines for adhesive anchors, such as EAD-00-0601 [42]. No edge preparation of the drillings was performed. A precured installation technique was employed for the anchors, which controlled the impregnated length so that it did not exceed two-thirds of the embedded depth, following the same procedure described in [20].

### 2.3. Instrumentation and Loading

The three-point bending test was chosen in this research because its results are easily applied to real beams, and to allow comparison with other experimental studies, such as [31]. For the tests, the beams were located on a steel frame that emulated the situation of simply supported beams.

The FRP reinforcements and anchors were monitored by means of attaching strain gauges to the external layer of the reinforcements and to the anchor dowels. The location of the gauges can be seen in Figure 4. Omega^®^ KFH-10-120-C1-11L1M2R strain gauges were employed in the research, due to their grid size (10 mm). The extensometer box was calibrated for the strain gauge factor (2), for the nominal resistance (120 Ω), and for the configuration at quarter Wheatstone bridge. No relevant temperature variations appeared during the tests, which were conducted in a controlled room of a laboratory, and, consequently, no compensation was required for temperature adjustment of monitorization with the strain gauges.

It should be noted that the monitorization of internal stresses serves for better identification of the contribution of the anchor to the overall performance at all times, by means of producing an independent load-strain curve for the reinforcement and the anchor dowel. It is noteworthy that monitorization of internal stresses in the anchor aimed at better identifying the contribution of the anchor to overall performance prior to debonding and as debonding progressed. Strain gauges were attached to the dowel region of the anchor prior to its insertion in the drilling, after the complete hardening of the matrix of the anchor dowel. The alignment of the strain gauges was controlled to guarantee that it corresponded to the longitudinal direction of the anchor.

**Figure 5:**
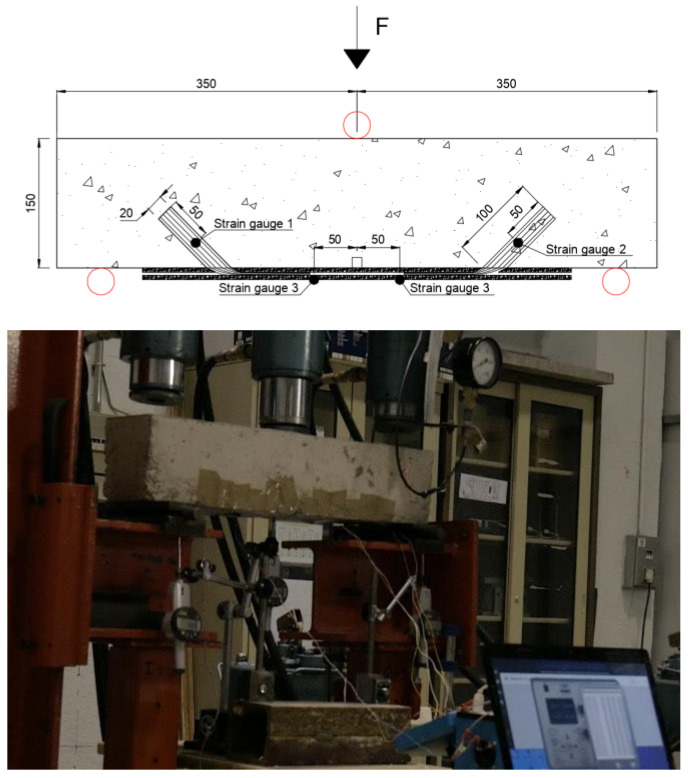
Test set up and instrumentation arrangement.

The load was applied through a hydraulic jacket, attached to a manometer that allows consulting intermediated loads. Intermediate values for load–displacement control were obtained every 1.25 kN, for assessment of proper measurement of the strain gauges as internal monitorization. The load was introduced in sustained steps of 5 kN, with continuous monitorization of the midspan deflection, and of the strains in the reinforcement and anchors. Load speed allowed intermediate measurement of deflections and load every 1.25 kN. The steps of 5 kN were employed to stop introducing load until the strain gauges were stabilized per each load step. The test set up is collected in Figure 5.

### 2.4. Unanchored Beam Strength Prediction

The ultimate strength for control series (unanchored joints) was estimated by calculating the bond strength according to the model for inter-crack debonding proposed by fib90 bulletin [8]. This model was adopted due to its easy of application, its basis on fracture mechanics and its calibration through 214 experimental results of beams [8]. Then, the sectional equilibrium method of force and bending moment described in the same bulletin was employed for the estimation of the ultimate load of the reinforced beam, under two considerations: the rupture strain of the FRP reinforcement, and the debonding strain corresponding to inter-crack debonding.

The value corresponding to FRP rupture was fixed as a theoretical maximum for comparison of material exploitation with the FRP anchors. Additionally, the load corresponding to debonding failure was calculated for comparison with the results of the control series. 

The rupture strain of the FRP reinforcement according to the average values in [34,35] for is 1.55 × 10^−2^. From this value it can be concluded that, if the rupture strain of the FRP is attained, the maximum strain in the concrete is 3.9 × 10^−3^, which is close but higher than the compressive strain of the fib90; in this case, in which concrete crushing is likely to occur and if we limit the strain in the concrete the normative value (3.5 × 10^−3^), the ultimate load would be 90.4 kN. The sectional equilibrium in this case would result in an ultimate load for debonding failure of 47.8 kN. 

## 3. Results and Discussion

A total of 10 beam tests were conducted to evaluate the impact of the bonded length behind the spike anchors, and to isolate the behaviour in the anchor fan-to-sheet interfaces when the reinforcement is unbonded. The results of three different positions of the anchor (B series) and of unbonded anchored reinforcements (U) were compared to a control series of bonded, unanchored reinforcements (C). The results are collected in Table 2.

The response of each specimen in terms of load-stress response is collected in Figure 3, in which the calculated ultimate load for FRP rupture is also plotted.

The first observation is that concrete crushing (expected at a load of 90.4 kN) was not achieved in any specimen. This indicated that height of the beams were appropriated for the evaluated anchor and sheet arrangement, as high exploitation of concrete was obtained, but without compromising the hierarchy of failure modes. It should be noted that the desirable failure mode hierarchy in FRP-reinforced beams is as follows: i) debonding at the highest possible stress in the FRP sheet followed by ii) ductile behaviour with post-peak performance, promoted by the presence of anchors with anchor rupture (full exploitation of anchor), thus provoking iii) failure of the pre-cracked beam by loss of the tensile force of the sheet.

### 3.1. Failure Modes

**Figure 6:**
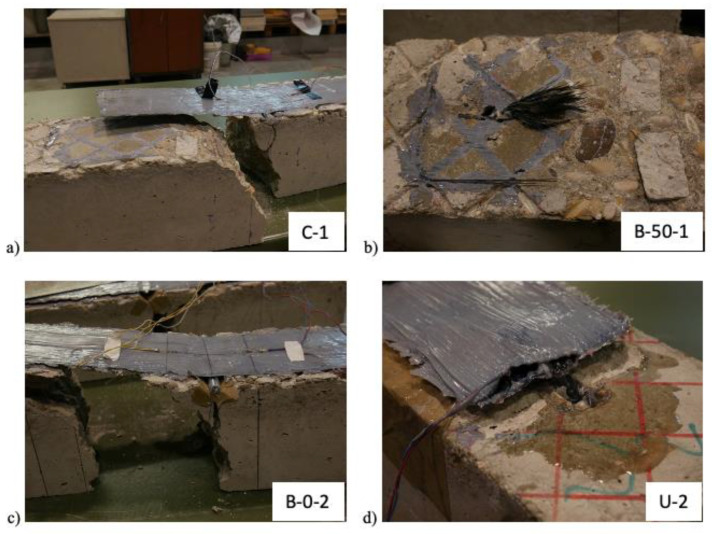
Observed failure modes: (**a**) Debonding; (**b**) Anchor rupture; (**c**) Concrete splitting; and (**d**) Anchor slippage.

As collected in Table 2, four prevailing failure modes were observed. These failure modes are presented in Figure 6.

All specimens with no anchor exhibited debonding failure, which occurred at several millimetres from the outer surface of concrete and provoked the shear rupture of the adhesive resin along the indentations.

In most bonded specimens with anchors, the failure mode was debonding followed by anchor rupture, which occurred in the bending zone or in the beginning of the anchor fan. In Figure 6b, it is possible to observed that the fibres are properly impregnated, exhibiting a rupture independent from the adherence to the FRP sheet. The failure provoked by concrete splitting is caused by the apparition of an almost horizontal cracking line that diverges from the main vertical bending cracking, thus provoking concrete separation and hinders the full exploitation of the anchor, as the beam splits in two parts by the propagation of the cracking up to the bonded surface.

The last failure mode, anchor slippage, occurred in unbonded specimens and implied that the test could not be completed up to the rupture of the anchor fibres. The test was stopped when the attained load decreased due to the slippage of the anchor in the drill. It is possible to observe from Figure 6d how there is a space between the sheet and the concrete surface. The slippage was so that the inner strain gauges attached to the anchor dowel arose in some of the specimens, without observable fibre rupture in the bending region. This premature failure mode may be attributed to the stress distribution in the beam in the moment when the anchor is first subjected to tensile stresses, which may affect the capacity in shear in the FRP-to-concrete interface of the drill.

### 3.2. Control Series

**Figure 7:**
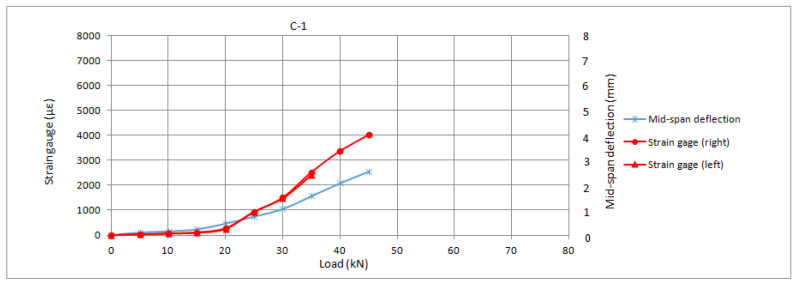
Load-slip response of the control series (C series).

The control series exhibited complete debonding of the reinforcement followed by concrete rupture. This is consistent with results from literature and constitutes the expected failure mode for unanchored reinforcements, in which tensile strength of the fibres cannot be attained due to premature debonding from the substrate. The preparation of the substrate prevented to a noticeable extent the cohesive failure within the first millimetres of the substrate, which was largely replaced by tangential failure of the adhesive. Notwithstanding, the debonding value did not significantly differ from the calculated one (the average value being 94% of the theoretical value according to the fib90), thus, it can be concluded that the preparation of the substrate allowed a proper development of the adherent strength according to the existing predictive models. The load-slip response of the control series is presented in Figure 7. 

It can be observed from the graphs presented in Figure 7 that the strain in the reinforcement is linear and almost negligible prior to the initiation of concrete cracking, which occurred at acting loads of around 20–25 kN. Once cracking of concrete is initiated, the reinforcements achieved an almost linear behaviour, but with higher stresses, up to the complete debonding when there is a sudden drop in the strains.

### 3.3. Bonded, Anchored Joints

The bonded, anchored joints (B series) exhibited a common failure for anchor rupture due to stress concentration in the bending region. In these series, it is possible to observe differences in behaviour depending on the relative position of the anchor within the bonded length.

**Figure 8:**
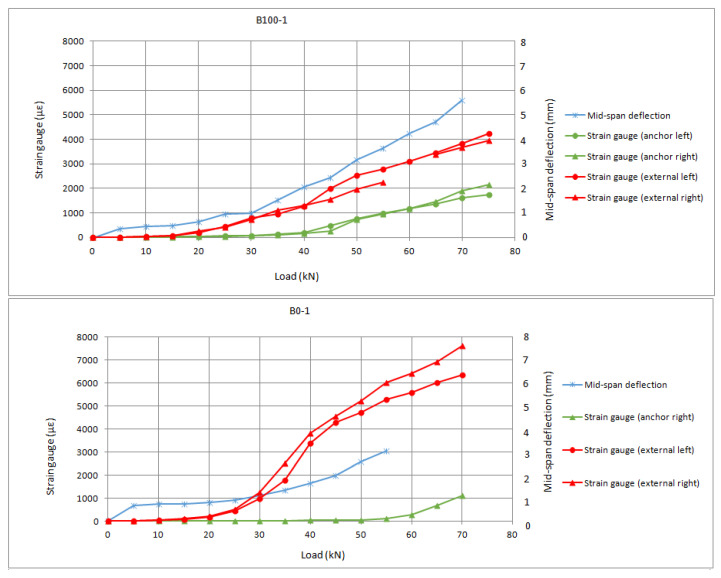
Load-slip response of the B series.

In the literature, shorter bonded lengths behind the anchor have been related to lower utilization of the anchor’s properties. In Zhang et al. [25], it was concluded that the bonded length behind the anchor is much more influential that the total bonded length (in front of and behind the anchor). In Cortez et al. [29], an analytical model was proposed including the influence of the bonded length behind the anchor, reflecting that there is a direct relation between achievable force and bonded length behind the anchor. In Figure 8, the results from B series are presented. No proper measurement was obtained for the B-50-series, as the nodes failed before the cracking bending moment.

It can be observed from Figure 8, that there is a difference in terms of the load at which load-transfer to the anchors is initiated, as a result of the different position of the anchor. It should be noted that the anchor’s location closer to the loaded end (B-100 series) allows for fuller exploitation of the anchor as its contribution is fruitfully added to the remaining bond behaviour. In the B-series, the connector begins to carry load prior to complete debonding, so debonding stops propagating towards the end of the FRP sheet and thus provides a synergistic effect with the anchor. This can be contrasted with the greater slope in the B-0 series, in which, at the load corresponding to complete debonding of the control series (around 45 kN), there is a detachment of the FRP sheet in front of the anchor, and from 60 to 70 kN the load is almost completely carried by the anchors. 

### 3.4. Unbonded, Anchored Joints

The series was designed to reproduce the behaviour when bond loss has occurred, to isolate the anchor’s performance, and to calculate whether there is any by-effect of the adherence at initial loads steps. The objective was to evaluate to what extend the adherent mechanism is synergic with the anchorage systems in anchored, bonded FRP reinforcements.

Series evaluated without adherent mechanism (U series) exhibited a particular failure mode which consisted of anchor slippage at low loads. The tests were interrupted when slippage occurred, which hindered the full exploitation of the anchors, as the load could not be sustained in time. In this sense, no anchor rupture was observed in this series. With this premature failure mode, the maximum loads were considerably lower than those of the B-series. The results are in good agreement with the impact on the anchorage system of partial loss of bond previously observed by Sun [37], and with the tests by Eshwar et al. [43], as further discussed in Section 4.

**Figure 9:**
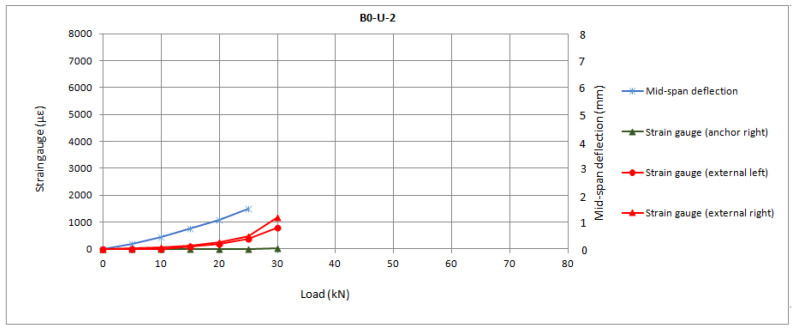
Load-slip response of the U series.

In Figure 9, the load-slip responses of the U series are presented.

It can be observed from Figure 9 that the strain in the anchors prior to the premature slippage is much lower than the ultimate strain prior to anchor rupture as registered in the B series. In fact, the measurements for both reinforcement and anchor up to 30 kN in the U-2 specimen are comparable to the results of all specimens from the B series. The difference is that, at that point, the reinforcement is unstressed as a consequence of the slippage, instead of being further stressed up to the debonding strain (in which the anchors begin to transfer the load). This may imply that the stress distribution around the drill has affected the shear stresses of the embedded length of the anchor, hindering fuller use of the connector’s capacity.

All graphs from internal monitoring show the values per 5 kN step of load. This allows removing the strain gauges noise prior to stabilization at each sustained step. There was an almost linear behaviour among load steps, with changes in slope being clearly identifiable. This linearity was registered at every second in the strain gauges. In Figure 7, Figure 8 and Figure 9, the progressive increase in strains in the FRP sheets was recorded from cracking of concrete (which started at step of load 20 kN). A change of slope in the load–strain relationship of reinforcement sheets was generally observed as strains increase in FRP anchors. It is possible to establish an approximate initial load for the anchor’s contribution at step 45 kN, which corresponds to debonding propagation of the control series. However, it was impossible to accurately define this point and differences between series as some of the inner strain gauges attached to the anchor dowels failed to register strains. This limitation indicates that, given the sensibility of the strain gauges, for future works it is convenient to attach at least three strain gauges per anchor. 

## 4. Assessment of Results by Comparison with Existing Models and Available Tests

Following Cortez et al.’s approach [29], the behaviour of the bonded, anchored joints was calculated as the debonding force plus the contribution of the anchor, which may depend on the configuration of the anchor (that is, of its ultimate load), and of the bonded lengths in front of the anchor and behind it. For the evaluated specimens, given that the U series presented slippage failure, the ultimate load was calculated according to Kim and Smith’s model for adherent failure of anchors [22]. A value of 34 kN was estimated for the slippage of the anchors, which is consistent with the results of this series being, respectively, 24.6 kN and 38.2 kN. This value for adherent or mixed failure can be taken as a reference value for the connector’s strength, despite the fact that some of the specimens in the U series exhibited concrete cone failure plus fibre rupture. This can be explained by the anchors having been designed for developing the optimal behaviour, with the failure in the border between the two failure modes (adherent failure and fibre rupture in the bending region).

For the B series, there are slight differences in the positive impact of the bonded length behind the anchor. This may be caused by the bonded lengths that were installed in front of the anchors, the model having been calibrated against tests in which the bonded length in front of the anchor is lower than the effective bond length when there exists bonded length behind the anchors. According to the results of the unbonded series, and of the control series, it is possible to estimate the exploitation of the anchors when a sufficient bonded length is provided behind the anchor and/or in front of it. The results are collected in Table 3, in which P_anc_ was the calculated value for the connector’s failure, PC-series is the average evaluated force of the control series, P_U-series_ is the average evaluated force of the U series, 31.4 kN, and P_db_ is the calculated value for the debonding force in the reinforcement. In the table, values greater than 1 for (P − P_db_)/P_anc_ indicate the existence of a synergistic effect between the adherent mechanism and the connector’s behaviour.

According to Table 3, the synergistic effect occurs to half of the specimens belonging to B-100 and B-0 series, which achieves greater loads than the expected addition of bond strength plus anchor’s capacity, according to the evaluated values. Furthermore, all specimens belonging to these same series are achieving greater loads than the expected addition of bond strength plus anchor’s capacity, according to the predictions. Additionally, from Table 3, it can be observed that the tensile force in the reinforcements in all the bonded, anchored specimens is close to the ultimate tensile force, according to the average value of the tensile strength of the sheets. From this it can be inferred that not only do the anchors have fuller use in conjunction with the adherence behaviour, but also that the installation of anchors in bonded joints can lead to fuller use of the FRP reinforcement sheets in concrete specimens.

Test results have also been compared with previous beam tests by Sun et al. [37], in which the studied beams have similar geometry to the one evaluated in this study. It should be noted that the purpose of the research was to evaluate the enhancement provided by patches as an additional system to the spike anchors, comparing completely bonded and partially unbonded sheets. The main difference with current research is that, in this paper, completely unbonded sheets were used for comparison, and anchor individual performance was optimized through dowel angle plus insertion of the anchor between two FRP sheets. The bonded length in the U-TLP-R13-F49 series was limited to the dimensions of the patch, which cover the area of the anchor fan plus the length behind the anchor’s insertion point. Results by Sun et al. [37] are collected in Table 4 employing the parameters for exploitation from Table 3 and are discussed below.

From Table 4, it can be observed that the reinforcement sheet employed by Sun et al. [37], which had a mean tensile strength of 1052, is more likely to suffer tensile FRP rupture as it was observed in some of the specimens (which exhibited what the authors called “sheet fracture”). On the other hand, the arrangement of the anchors does not allow full exploitation of the anchor, the high ultimate loads attained being mostly provided by the adherent behaviour. In this sense, it is necessary to indicate that the authors employed thicknesses of sheets of 0.51 mm in one layer, which is 1.97 times the total thickness (for two layers) in present research. The results by Sun et al. in terms of exploitation of FRP sheet tensile properties is noticeably high according to the performed analysis. This can be explained by the arrangement of the patches in series B-TP-R13-F49, B-TLP-R13-F49, and U-TLP-R13-F49, which is indeed related to the arrangement between the two sheets in present research. It can be concluded that, when the anchor is located onto the only layer of FRP sheet, delamination between the anchor and the reinforcement can occur, as previously observed by [14] and is also found in B-NP-R13-F49. On the other hand, the solution provided in [37] with patch anchors has the effect of providing greater stiffness (leading to sheet exploitations higher than 1 in B-TLP-R13-F49 series), while ensuring proper load-transfer mechanism between the anchor and the sheet. This effect was similar to present research, in which the anchors were located between the two layers in all specimens. Another remarkable observation is that U-TLP-R13-F49, with a bonded length in front of the anchor equal to the connector’s fan length, obtains worse results than the equivalent completely bonded sheets (B-TLP-R13-F49).

## 5. Conclusions

Based on the experimental results, the following conclusions can be drawn:

1. FRP anchors enhance joint behaviour, with anchored FRP reinforcement sheets bonded to concrete beams, reaching peak loads up to 155% of the ones of the control series.
2. The overall performance of the FRP anchored sheets seems to depend on the bonded length in front of the anchor and behind it. The best result was obtained with the anchor located closest to the midspan.
3. The failure mode of the beams depends on whether there exists a bonded length. The beams with no bonded length exhibited slippage of the anchor, while all beams with bonded length presented fibre rupture in the bending area.
4. There exists a synergistic effect of the contribution of the adherent mechanism and of the anchors to the overall strength of the flexural reinforced beams. The overall strength in bonded, anchored plates can be expressed as the addition of the bond strength plus the connector’s contribution, which is a fraction of the connector’s capacity.
5. The installation of spike anchors in conjunction with the adherent mechanism allows fuller use of the sheets in concrete beams.
6. The adherent performance of the FRP-to-FRP did not limit the anchor’s contribution to bond strength.

The eventual influence of the internal stirrups, which could not be fully addressed in the paper, may the object of future works, such as FEM modelling of the beams, to further study the possible effects of the confined concrete. Additionally, it should be noted that the tests are subjected to scatter due to the heterogeneity of the concrete material in specimens of the given size, with a greater influence of the aggregate diameter than that found in construction beams. Another limitation of the study is caused by the selection of the beam size. In this sense, additional research is required for concrete beams with greater height, in which equilibrium with FRP sheet is achieved with smaller compressive zone regions in the concrete.

## Figures and Tables

**Table 1:** Test matrix.

Series	Anchors	Bonded Length in Front of the Anchor	Bonded Length behind the Anchor
Control	-	-	-
B-100	1	150	100
B-50	1	200	50
B-0	1	250	0
U-0	1	0	0

**Table 2:** Test results.

Specimen	Ultimate Step Load (Ultimate Load *) (kN)	Average Ultimate Sustained Load (kN)	Pult/Pcontrol	Pult/Pdb **	Failure Mode
C-1	50 (50)	45	1.11	1.05	Debonding
C-2	40 (42.5)		0.89	0.84	Debonding
B-100-1	75 (75)	70	1.67	1.57	Anchor rupture
B-100-2	65 (67.5)		1.44	1.36	Anchor rupture
B-50-1	60 (60)	60	1.33	1.25	Anchor rupture
B-50-2	60 (61.25)		1.33	1.25	Anchor rupture
B-0-1	70 (70)	67.5	1.55	1.46	Anchor rupture
B-0-2	65 (65)		1.44	1.36	Concrete cracking
U-1	20 (20)	25	0.44	0.42	Anchor slippage
U-2	30 (31.25)		0.67	0.63	Anchor slippage

* The bracketed number corresponds to rupture load, when at least 1.25 kN was applied to the specimen before failure following the last sustained step of load. ** Pdb was calculated according to the model for IC debonding of fib90, with average values. The stress in the reinforcement was estimated according to the predicted ultimate load for the control series using the model in [8], so Pult/Pdb is the result of dividing the experimental ultimate load by the predicted ultimate load for unanchored joints.

**Table 3:** Exploitation of sheets and connectors in bonded, anchored beams.

Specimen	Ultimate Force P_u_ (kN)	(P_u_ − P_C-series_)/P_U-series_	(P_u_ − P_db_)/P_anc_	% Tensile Strength of Reinforcement *
B-100-1	102.8	1.39	1.54	0.96 (1.14)
B-100-2	88.1	0.93	1.11	0.82 (0.98)
B-50-1	80.8	0.69	0.90	0.76 (0.89)
B-50-2	80.8	0.69	0.90	0.76 (0.89)
B-0-1	95.4	1.16	1.33	0.89 (1.06)
B-0-2	88.1	0.93	1.11	0.82 (0.98)

* Values according to average material properties in [39,40], values in brackets according to nominal characteristic values from product datasheet.

**Table 4:** Exploitation of sheets and connectors according to results in Sun et al. [37].

Series	Ultimate Force P_u_ (kN)	(P_u_ − P_db_)/P_anc_	% Tensile Strength of Reinforcement
B-NP-R13-F49	57	0.05	0.76
B-TP-R13-F49	67	0.33	0.89
B-TLP-R13-F49	85	0.84	1.15
U-TLP-R13-F49	74	0.53	0.99

## Data Availability

Raw data will be made available by e-mail contact to corresponding author.
